# The Small RNA ErsA of *Pseudomonas aeruginosa* Contributes to Biofilm Development and Motility through Post-transcriptional Modulation of AmrZ

**DOI:** 10.3389/fmicb.2018.00238

**Published:** 2018-02-15

**Authors:** Marilena Falcone, Silvia Ferrara, Elio Rossi, Helle K. Johansen, Søren Molin, Giovanni Bertoni

**Affiliations:** ^1^Dipartimento di Bioscienze, Università degli Studi di Milano, Milan, Italy; ^2^Department of Clinical Microbiology, Rigshospitalet, Copenhagen, Denmark; ^3^Department of Clinical Medicine, Faculty of Health and Medical Sciences, University of Copenhagen, Copenhagen, Denmark; ^4^Novo Nordisk Foundation Center for Biosustainability, Technical University of Denmark, Kongens Lyngby, Denmark

**Keywords:** *Pseudomonas aeruginosa*, small regulatory RNA, post-transcriptional regulation, biofilm, virulence

## Abstract

The small RNA ErsA of *Pseudomonas aeruginosa* was previously suggested to be involved in biofilm formation via negative post-transcriptional regulation of the *algC* gene that encodes the virulence-associated enzyme AlgC, which provides sugar precursors for the synthesis of several polysaccharides. In this study, we show that a knock-out *ersA* mutant strain forms a flat and uniform biofilm, not characterized by mushroom-multicellular structures typical of a mature biofilm. Conversely, the knock-out mutant strain showed enhanced swarming and twitching motilities. To assess the influence of ErsA on the *P. aeruginosa* transcriptome, we performed RNA-seq experiments comparing the knock-out mutant with the wild-type. More than 160 genes were found differentially expressed in the knock-out mutant. Parts of these genes, important for biofilm formation and motility regulation, are known to belong also to the AmrZ transcriptional regulator regulon. Here, we show that ErsA binds *in vitro* and positively regulates *amrZ* mRNA at post-transcriptional level *in vivo* suggesting an interesting contribution of the ErsA-*amrZ* mRNA interaction in biofilm development at several regulatory levels.

## Introduction

Biofilm formation is considered to be an adaptive strategy of the human pathogen *Pseudomonas aeruginosa*, and the switch from the motile to a sessile mode of growth represents an important step in the virulence of this pathogen (Costerton et al., 1999).

Biofilms are microbial communities assembled in a self-produced matrix of exopolysaccharides, proteins and DNA (Ma et al., 2006), generating conditions that confer resistance and protection against antimicrobial agents and the immune system. The biofilm lifestyle cycle of *P. aeruginosa* PAO1 develops through coordinated stages. Adhesion to a surface is the first step in the colonization of *P. aeruginosa* and is followed by cell-to-cell aggregation. Attachment is an irreversible condition characterized by formation of microcolonies that develop in structured and three-dimensional clusters. During these two stages, the bacterial cells display three types of motility: swimming movement in liquid or low-viscosity conditions, swarming on semisolid surface and twitching on a solid surface. Swarming motility is based on flagella and type IV pili as well as on biosurfactants, swimming is flagella-dependent, and twitching relies on extension and retraction of type IV pili (O’Toole and Kolter, 1998; Kohler et al., 2000; Wang et al., 2014). The final stage of biofilm development is bacterial dispersion, in which the bacteria re-enter the planktonic state, spreading and colonizing other surfaces (Flemming et al., 2007; Wang et al., 2014).

**FIGURE 1:**
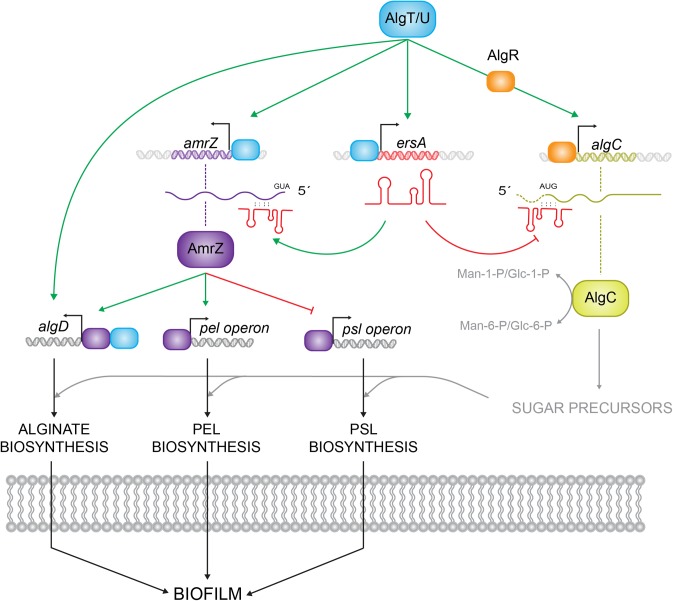
Schematic representation of different levels of AlgU-dependent regulatory routes in *Pseudomonas aeruginosa*. AlgU drives the expression of the alginate biosynthetic operon by activating the expression of *algD* promoter and it modulates exopolysaccharides (Pel and Psl) production by inducing the expression of transcriptional regulators, as AmrZ and AlgR, and the small RNA ErsA, which regulates *algC* at the post-transcriptional level. Green arrows represent positive regulation, red arrows negative regulation.

As summarized in **Figure 1**, intertwined regulatory pathways and numerous regulators control transcriptionally and post-transcriptionally biofilm development. Most of these regulators are coordinated by the alternative sigma factor AlgT/U (σ^22^) (Potvin et al., 2008), a mediator of stress response and a functional homolog of *Escherichia coli* σ^E^ (Yu et al., 1995). AlgU regulates alginate production driving the expression of *algD* operon, and activating two transcriptional regulators, AlgR and AmrZ, both required for alginate production in multiple mucoid strains (Mohr et al., 1991, 1992; Yu et al., 1995). AmrZ, besides the interaction with *algD*, also directly affects the *P. aeruginosa* exopolysaccharides profile. In fact, as shown in **Figure 1**, AmrZ triggers the expression of the exopolysaccharide Pel interacting with a member of the *pel* operon (*pelB*) and represses the expression of the exopolysaccharide Psl binding to the *pslA* promoter. In addition, AmrZ affects the intracellular levels of the signaling molecule bis (3′–5′)-cyclic diguanylic monophosphate (c-di-GMP) (Jones et al., 2014; Petrova et al., 2014; Xu et al., 2016). Pel and Psl exopolysaccharides are the major contributors to *P. aeruginosa* biofilm structure and development. Psl supports the cell-to-cell interactions during the initial attachment and adhesion phase, forming a fiber web to constitute a scaffold for the biofilm shaping, and Pel provides structural stability to the global configuration (Ma et al., 2006, 2007; Yang et al., 2011; Jennings et al., 2015).

Biosynthesis of Pel, Psl, and LPS uses common sugar precursors supplied by the AlgU-induced AlgC enzyme, which coordinates the levels of exopolysaccharides in the cell, catalyzing the conversion of Man-6-P and glucose-6-P (Glc-6-P) to Man-1-P and Glc-1-P, respectively (Coyne et al., 1994; Ma et al., 2012). AlgC is positively regulated by AlgR at the transcriptional level, and negatively regulated by the small RNA (sRNA) ErsA at the post-transcriptional level (Zielinski et al., 1991; Ferrara et al., 2015). ErsA is a novel sRNA recently characterized in *P. aeruginosa* whose expression responds to several infection cues such as limited iron availability, temperature shifts from environmental to body temperature and reduced oxygen conditions. The incoherent feed-forward loop settled by ErsA and AlgU to fine-regulate AlgC was supposed to be an additional regulatory route in the complex process of biofilm shaping, in particular balancing the sugar precursors production in the exopolysaccharides biosynthesis (Ferrara et al., 2015).

In a recent study (Zhang et al., 2017), ErsA has been described to bind and regulate at the post-transcriptional level *oprD* mRNA, coding for a porin which highly contributes to carbapenems sensitivity. The overexpression of ErsA negatively affects translation of *oprD* mRNA and consequently the OprD protein level, reducing susceptibility to meropenem treatment. These findings contribute to enforce the role of ErsA in *P. aeruginosa* pathogenesis by regulating different virulence traits.

sRNAs can regulate multiple targets, allowing the cells to have a fast response to stress conditions and adapt in a short time frame to environmental changes (Beisel and Storz, 2010).

ErsA provides a relevant regulatory contribution balancing metabolism and virulence routes by regulating the checkpoint enzyme AlgC and it was conceivable to hypothesize novel ErsA targets in the large landscape of regulatory routes connected to exopolysaccharides production and biofilm formation.

In this study, we scrutinized for the first time the regulatory pattern of ErsA in *P. aeruginosa* biofilm formation revealing a positive contribution of the sRNA to biofilm maturation and shaping. An RNA-seq approach allowed us to identify several genes involved in this process, whose expression was deregulated in an ErsA deletion mutant. Most of these genes belong to AmrZ regulon, which was shown to be a novel direct target for ErsA (**Figure 1**).

## Materials and Methods

### Bacterial Strains and Media

Bacteria and plasmids used in this study are listed in Supplementary Table S1. *E. coli* strains were grown at 37°C in Lysogeny Broth (LB). *P. aeruginosa* strains were grown at 37°C in LB or in Brain Heart Infusion Broth (BHI) or Artificial Sputum Medium (ASM) in flasks at 200 r.p.m.. When required, for *E. coli* strains the media were supplemented with 10 μg/ml gentamycin, 100 μg/ml ampicillin, 25 μg/ml kanamycin, and for *P. aeruginosa* strains with 50 μg/ml gentamycin and 300 μg/ml carbenicillin. For monitoring biofilm development in flow-chambers conditions, PAO1 wild-type and PAO1 Δ*ersA* (Ferrara et al., 2015) were chromosomally tagged with green fluorescent protein (GFP) and grown in modified FAB medium (Heydorn et al., 2000) supplemented with 0.3 mM glucose.

ErsA overexpression was obtained from pGM-*ersA* plasmid (Ferrara et al., 2015) using arabinose 0.2% when required.

### Plasmid Construction and Mutant Generation

Oligonucleotides used in this study are listed in Supplementary Table S2. Translational fusions pBBR1 *amrZ::sfGFP*, *amrZ*CIS1::*sfGFP, amrZ*ΔIS2::*sfGFP and amrZ*CIS1ΔIS2::*sfGFP* under the *P*_*LtetO*-1_ constitutive promoter were generated as follows. A DNA fragment of 161 bp including 56 nt of UTR-region and 35 codons of the open reading frame (ORF) of *amrZ* was amplified by PCR with oligos 1/2 (Supplementary Table S2), digested with *Nsi*I-*Nhe*I and cloned into the *sfGFP* reporter vectors pXG10-SF resulting in the plasmid pXG10-*amrZ::sfGFP.* Likewise for *amrZ::sfGFP*, 161 bp including 56 nt of UTR-region and 35 codons of the ORF of *amrZ* were amplified by PCR with oligos 1/2 (Supplementary Table S2) from pUCIDT *amrZ*CIS plasmid, carrying the synthetic and modified sequence of *amrZ*, digested with *Nsi*I-*Nhe*I and cloned into pXG10-SF to generate the translational fusion pXG10-*amrZ*CIS1::*sfGFP*. The translational fusion pXG10-*amrZ*ΔIS2::*sfGFP* was generated amplifying a fragment of 119 bp including 56 nt of UTR-region and 21 codons of the *amrZ* ORF with oligos 1/3 (Supplementary Table S2) digested with *Nsi*I-*Nhe*I and cloned into pXG10-SF. *AmrZ*CIS1ΔIS2::*sfGFP* was constructed amplifying a fragment of 119 bp including 56 nt of UTR-region and 21 codons of the *amrZ* ORF with oligos 1/3 (Supplementary Table S2) from pUCIDT *amrZ*CIS plasmid. All the fragments from the *P*_*LtetO*-1_ promoter to the end of the GFP reporter gene, including the different versions of *amrZ*, were amplified from pXG10-*amrZ::sfGF, amrZ*CIS1::*sfGFP, amrZ*ΔIS2::*sfGFP* and *amrZ*CIS1ΔIS2::*sfGFP*, using oligos 9/10, digested with *Cla*I-*Xba*I and cloned into the low-copy number shuttle vector pBBR1-MCS5 generating the pBBR1-*amrZ::sfGFP, amrZ*CIS1::*sfGFP, amrZ*ΔIS2::*sfGFP and amrZ*CIS1ΔIS2::*sfGFP*, respectively. All the plasmids were then transformed into *P. aeruginosa* strains as reported previously (Ferrara et al., 2015).

### Mini-Tn7-*gfp* Strain Construction

A PrrB1-*gfp*-a transposon cassette was inserted into the chromosome of PAO1 wild-type and Δ*ersA* by conjugation using pBK-miniTn7-ΩGm as a delivery plasmid carrying the cassette inserted into *Not*I site as reported previously (Lambertsen et al., 2004).

### Biofilm Adhesion in 96-Wells Peg-Lid Microtiter

A quantity of 200 μl of overnight bacterial cultures grown in BHI or ASM and diluted to OD_600_ = 0.01, with the addition of carbenicillin 300 μg/ml and arabinose 0.2% when required, was aliquoted into 96-well peg-lid microtiter plates (Nunclon Delta Surface Cat. No.167008, Nunc TSP Cat. No.445497, Thermo Scientific) as reported previously (Harrison et al., 2010). The plates were incubated at 37°C in aerobic conditions with 100 r.p.m. stirring. After 20 h of incubation, growth was monitored by measuring the OD_600_, and the ability of the *P. aeruginosa* strains to adhere to the polystyrene peg-lid was tested by crystal violet staining. Briefly, the peg-lid was washed twice with saline solution and then stained with 0.1% crystal violet for 20 min (O’Toole, 2011). Excess of stain was rinsed off by placing the peg-lid in saline solution before to solubilize the dye in absolute ethanol. The optical density of each well was measured at 590 nm. Biofilm formation was expressed in adhesion units as the result of the OD_590_/OD_600_ ratio and statistical analysis were performed using *T*-Test.

### Biofilm Development in Flow-Cells System

Biofilms were grown at 30°C in flow chambers composed of three individual channels as described previously (Møller et al., 1998). PAO1 wild-type and Δ*ersA* overnight cultures diluted to OD_600_ = 0.01 were inoculated into each flow channel with a small syringe. After 1 h without flow, each channel was supplied with a flow of 3 ml/h of FAB medium with glucose 0.3 mM, using a Watson Marlow 205S peristaltic pump. The mean flow velocity in the flow cells was 0.2 mm/s.

### Confocal Laser Scanning Microscopy and Image Processing

The microscopic analyses were performed using a Zeiss LSM510 confocal laser scanning microscope (CLSM; Carl Zeiss, Jena, Germany) equipped with an Ar/Kr laser and filter sets for GFP detection (excitation, 488 nm; emission, 517 nm). Images were obtained using a 40×/1.3 Plan-Neofluar oil objective.

Simulated shadow projection images and cross sections were generated using the IMARIS software package (Bitplane AG, Zürich, Switzerland).

The experiment was performed in triplicate for each strain acquiring seven random images for each channel every day for 3 days. Thus, 21 images for each time point were employed for the statistical analyses using COMSTAT 2.1 software^1^ (Heydorn et al., 2000; Vorregaard, 2008).

### Co-twitching and Co-swarming Motility Assays

Swarming assays were performed using Nutrient Broth (Nutrient Broth n°2 Oxoid) medium plates supplemented with 0.5% glucose and 0.5% Bacto-agar (Difco). Overnight cultures normalized at the same OD_600_ of PAO1 wild-type and Δ*ersA* were spotted on the same plate suitably spaced each other and placed at both 28°C and 37°C for 24 h.

Twitching was performed on LB plates supplemented with 1% Bacto-agar (Difco). The inoculation was performed with a sterile toothpick dipped in the overnight cultures and followed at 37°C for 24 h. Statistical analysis was performed on three independent replicates with GraphPad Prism software.

### RNA Sequencing and Data Analysis

For RNA-Seq, cultures of wild-type PAO1 and Δ*ersA* strains were grown to early stationary phase (OD_600_ = 2.7) in BHI medium. For each strain, total RNA was extracted from at least two independent biological replicates using Trizol reagent (Thermo Fisher Scientific Inc.) followed by RNA clean and concentrator kit (Zymo Research, Irvin, CA, United States) accordingly to vendors’ protocols. RNA quality was checked using RNA Nano kit on an Agilent Bioanalyzer 2100 machine. Samples with an RNA integrity number (RIN) greater than 9 were used in downstream analysis. Strand-specific sequencing libraries were prepared using 50 ng of mRNA-enriched samples as input for TruSeq stranded mRNA library preparation kit (Illumina) following vendor’s recommendations. Sequencing was performed on an Illumina NextSeq 500 to a depth of 15–20 million reads per sample. After quality filtering, raw reads were aligned using BWA aligner against *P. aeruginosa* PAO1 genome (NC_002516.2). Read count for gene relative abundance was obtained using HTSeq-count tool from HTSeq package (Anders et al., 2015), while differential expression analysis and statistical analysis were performed as previously described (Peano et al., 2014). RNA-seq data have been deposited in the ArrayExpress database at EMBL-EBI^2^ under accession number E-MTAB-6247.

### RNA Isolation and Synthesis

Total RNA was extracted as reported previously (Ferrara et al., 2012). RNA for RNA/RNA interaction assays was prepared by T7 RNA polymerase transcription of gel-purified DNA fragments. DNA fragments for ErsA RNA and *amrZ* mRNAs (*amrZ*, *amrZ*CIS1, *amrZ*ΔIS2, *amrZ*CIS1ΔIS2) preparations were amplified from *P.aeruginosa* PAO1 genomic DNA with oligo pairs 4/5 or 4/6 and 7/8, respectively. The transcription reactions were performed using the Riboprobe^®^ System-T7 (Promega) with 300 ng of DNA template. DNA probe was 5′-end–labeled with (γ-^32^P) ATP and T4 polynucleotide kinase (Promega) according to manufacturer’s instruction. Synthesized RNA was precipitated and resuspended in diethylpyrocarbonate-treated water. Purified RNA was checked by denaturing polyacrylamide gel electrophoresis and quantified using a Qubit Fluorometer.

### *In Vitro* and *in Vivo* Assays of sRNA/mRNA Interactions

To assess the ErsA/*amrZ* mRNA interactions *in vitro*, the binding reactions were set up as described previously (Ferrara et al., 2015). After the electrophoresis, the membrane was UV-crosslinked and hybridized with a [^32^P]-labeled oligo and the radioactive bands were acquired using a Typhoon^TM^ 8600 variable mode imager scanner (GE Healthcare BioSciences) and visualized with ImageQuant software (Molecular Dynamics).

Non-radioactive EMSA were performed using Mini-Protean^®^ Electrophoresis System (Bio-Rad) at 4°C and 150 V for 45 min. The gel was stained in SYBR^TM^ Gold Nucleic Acid Gel Stain diluted in 0.5 × TBE. Images were acquired by Gel Doc^TM^ XR+ (Bio-Rad) imaging system.

Fluorescence measurements of *P. aeruginosa* strains carrying the reporters pBBR1-*amrZ::gfp* were carried out as previously reported (Ferrara et al., 2015). Abs_595_ and fluorescence polarization FP_485/535_ were measured in a Tecan Infinity PRO 200 reader, using Magellan as data analysis software (Tecan). GFP activities were expressed in Arbitrary Units (AU) as ratio FP_485/535_/Abs_595_. Statistical analysis performed on three individual clones per strain using *T*-test.

## Results

### ErsA Is Required for Biofilm Adhesion and Development

**FIGURE 2:**
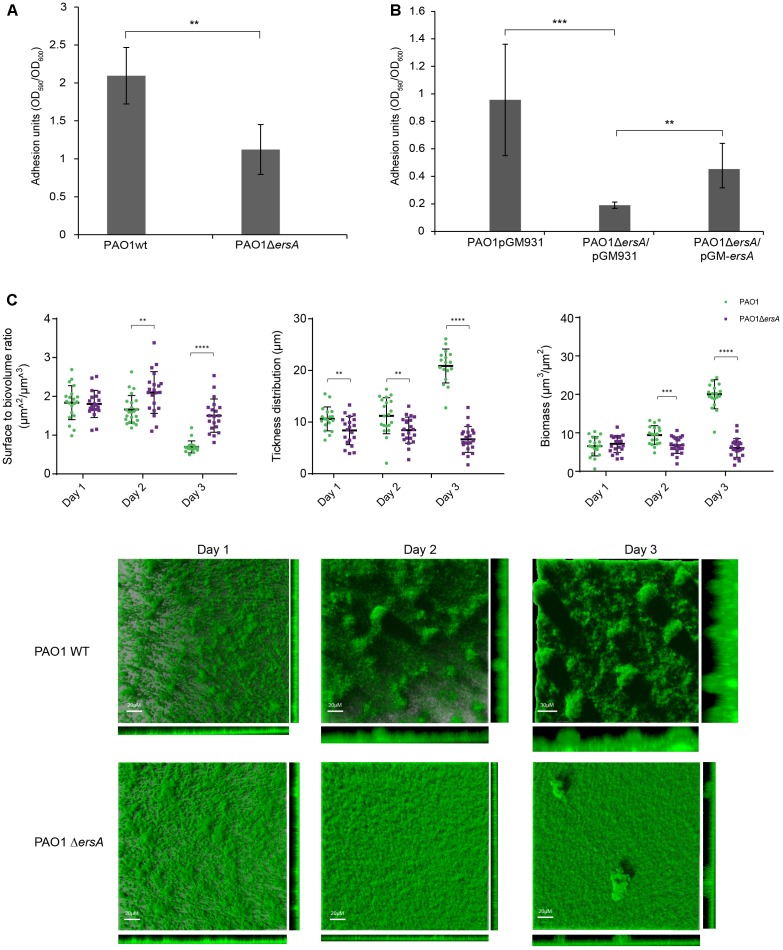
Biofilm formation of PAO1 wild-type, Δ*ersA*, wild-type/pGM931, Δ*ersA*/pGM931 and Δ*ersA*/pGM-*ersA* strains. **(A)** PAO1 *ersA* mutant strain produces less biofilm in BHI medium when compared to the wild-type strain. **(B)** The phenotype is rescued when the *ersA* mutation is complemented by the pGM-*ersA* plasmid (four replicates for each strain, 24 h at 37°C. Adhesion units are expressed as the ratio of biofilm formation optical density OD_590_ normalized for the bacterial growth OD_600_). *T*-Test, ^∗∗∗^*p*-value < 0.001, ^∗∗^*p* < 0.01, ^∗^*p* < 0.1. **(C)** Spatial distribution of 3 days-old flow-chamber-grown biofilms of PAO1 wild-type and Δ*ersA* GFP-tagged strains. The larger central plots are simulated fluorescence projections, in which long shadows indicate large, high micro-colonies. The scale bars shown are also valid for the right and lower frames. Surface to volume ratio, thickness distribution and biomass of PAO1 wild-type and Δ*ersA* values are means of data from 21 image stacks (seven image stacks from three channels). The statistical analysis was performed using GraphPad Prism software (^∗∗^*p*-value < 0.01, ^∗∗∗∗^*p* < 0.001).

We investigated the effects of deleting the *ersA* gene on biofilm formation using a semi-quantitative microtiter “peg-lid” assay in Brain Heart Infusion medium (BHI). As shown in **Figure 2A**, the ErsA deletion resulted in decreased biofilm formation in BHI compared to PAO1 wild-type strain, and the complemented strain carrying the plasmid pGM-*ersA* produces more biofilm than the *ersA* deletion mutant strain carrying the pGM931 empty vector (**Figure 2B**).

To examine the role of ErsA in *P. aeruginosa* biofilm architecture development, we cultivated the PAO1 wild-type and the Δ*ersA* GFP-tagged strain, in flow-chambers continuously supplied with modified FAB medium supplemented with glucose. Biofilm development stages were followed and visualized daily for 3 days by Confocal Laser Microscopy (CLSM). In agreement with biofilm formation in the microtiter “peg-lid” assays in BHI medium, the PAO1 Δ*ersA* strain developed less biofilm biomass than the wild-type, which showed the mushroom-like structures typical of 3-days old *P. aeruginosa* biofilms in flow-cells system (**Figure 2C**). The statistically significant differences in biomass and spatial structure between PAO1 wild-type and Δ*ersA* biofilms were determined by the COMSTAT 2.1 software (Heydorn et al., 2000; Vorregaard, 2008) as represented in **Figure 2C**. We further noticed the positive influence of ErsA on adhesion and biofilm formation when overexpressed in PAO1 wild-type and Δ*ersA* strains, grown in ASM (Supplementary Figure S1), which is defined to reflect the chemical environment of CF lungs (Sriramulu et al., 2005; Haley et al., 2012).

### ErsA Negatively Regulates Swarming and Twitching Motility

**FIGURE 3:**
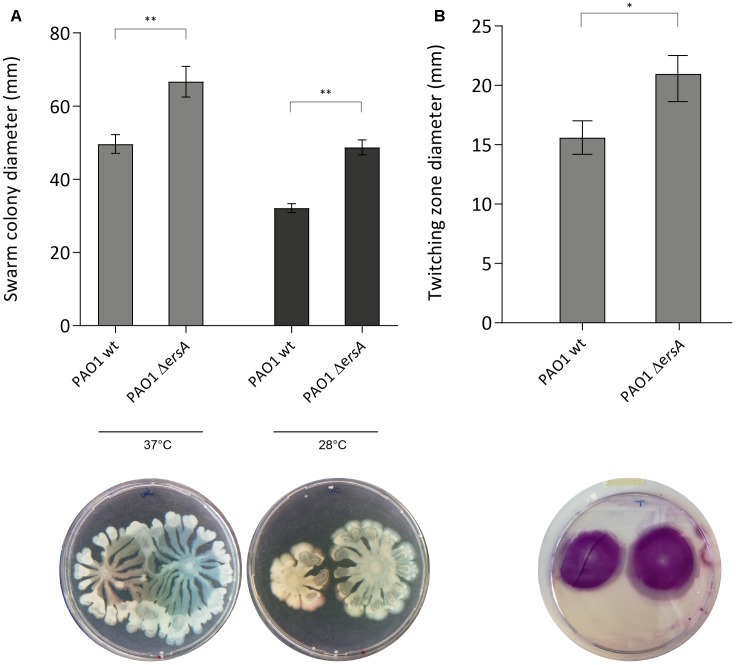
PAO1 and PAO1 Δ*ersA* motility. ErsA deletion results in more swarming motility compared to the PAO1 wild-type on 0.5% Nutrient Broth agar plates supplemented with 0.5% glucose at 37°C and 28°C **(A)**, and twitching motility at the plastic-1.0% LB agar interface stained with 0.1% crystal violet **(B)**. Statistical analysis was performed on three independent replicates with GraphPad Prism software (^∗^*p*-value < 0.05, ^∗∗^*p* < 0.01). The best representative pictures are displayed.

Motility is crucial in cell-to-cell adherence and attachment in early biofilm stages and it has been suggested an inverse regulation of motility and biofilm during biofilm development (Caiazza et al., 2007; Wang et al., 2014). Several transcriptional and post-transcriptional regulators are involved in these pathways and some of them coordinate both sessile and motile lifestyles (O’Toole and Kolter, 1998; Ramsey and Whiteley, 2004; Shrout et al., 2006; Gloag et al., 2013). To further investigate the involvement of ErsA on these biofilm-related phenotypes, we performed co-swarming, swimming and co-twitching experiments comparing PAO1 wild-type with Δ*ersA* strain. Our results reveal a negative influence of ErsA on both swarming and twitching motility (**Figure 3**) and the temperature conditions do not affect ErsA regulation on swarming motility (**Figure 3A**). No differences between PAO1 wild-type and Δ*ersA* mutant strain were observed for swimming motility (Supplementary Figure S2).

### ErsA Deletion Affects the Transcriptional Levels of 168 Genes in *P. aeruginosa* PAO1

Small RNAs are usually involved in post-transcriptional regulation, and the role of ErsA in biofilm development and motility shown in this study, suggested interference with the translation of transcriptional regulators as AmrZ. Thus, to expand the panel of ErsA targets in *P. aeruginosa* PAO1, and to have a better view of the effect of ErsA activity on the genome-wide gene expression, we performed an RNA-seq experiment comparing PAO1 wild-type to ErsA deletion mutant strains, grown to late exponential phase (OD_600_ of 2.7) in BHI medium. We observed 168 genes (Supplementary Table S3 and the most representative genes listed in **Table 1**) differentially expressed in the *ersA* deletion mutant when compared to the wild-type strain. Among the 29 genes upregulated in the *ersA* deletion mutant we identified genes involved in denitrification and nitrate metabolism (*narI, narJ, nirN*) as well as type VI and III secretion systems effectors (*tssA1, tsi4, tse6*).

**Table 1:** Selection of the most representative genes differentially expressed in PAO1 ErsA deletion mutant with Log_2_ (FC) ≤-1 or Log_2_ (FC) ≥ 1.

Locus tag	Name and description	Log2(FC)^†^	Fold change
*Down-regulated in PAO1*Δ*ersA strain*			
PA0105	*coxB*	-1,70889	-3,269092
PA0106	*coxA*	-1,82114	-3,5336031
PA0792	*prpD*, propionate catabolism	-3,035	-8,1964546
PA0795	*prpC*, citrate synthase 2	-2,68584	-6,4345978
PA1107**^‡^**	*roeA*, RoeA	-1,57126	-2,9716413
PA1596	*htpG*, heat shock protein HtpG	-1,69763	-3,2436766
PA2663**^‡^**	*ppyR*, Psl and pyoverdine operon regulator, PpyR	-2,18138	-4,5358722
PA3058**^‡^**	*pelG*, PelG	-1,4459	-2,7243272
PA3059**^‡^**	*pelF*, PelF	-2,06261	-4,1774136
PA3060**^‡^**	*pelE*, PelE	-1,89954	-3,7309422
PA3061**^‡^**	*pelD*, PelD	-1,54687	-2,9218255
PA3062**^‡^**	*pelC*, PelC	-1,75174	-3,3676448
PA3126	*ibpA*, heat-shock protein IbpA	-2,64174	-6,240839
PA3540**^‡^**	*algD*, GDP-mannose 6-dehydrogenase AlgD	-1,5095	-2,8471135
PA3877	*narK1*,nitrite extrusion protein 1	-3,06065	-8,3434844
PA3879	*narL*, two-component response regulator NarL	-1,67047	-3,1831828
PA4217	*phzS*, flavin-containing monooxygenase	-1,60294	-3,037617
PA4596	*esrC*, EsrC	-2,53876	-5,8108934
PA4760	*dnaJ*, DnaJ protein	-1,23304	-2,3506178
PA4761	*dnaK*, DnaK protein	-1,72393	-3,3033504
PA5053	*hslV*, heat shock protein HslV	-1,52351	-2,87491619
PA5054	*hslU*, heat shock protein HslU	-2,14174	-4,4129396
*Up-regulated in PAO1*Δ*ersA strain*			
PA0082	*tssA1*, TssA1	1,424308	2,6838574
PA0093	*tse6*, Tse6	1,500855	2,8301039
PA0509	*nirN*, NirN	2,22837	4,6860424
PA2775	*tsi4*, Tsi4	1,314079	2,4864355
PA3872	*narI*, respiratory nitrate reductase gamma chain	3,080462	8,4588527
PA3873	*narJ*, respiratory nitrate reductase delta chain	2,190598	4,5649467
PA3874	*narH*, respiratory nitrate reductase beta chain	1,653484	3,1459244


*^†^Log_*2*_ (FC) = log_*2*_ of fold change calculated as ratio between gene expression of PAO1 wild-type vs. PAO1 ΔersA. ^‡^Genes involved in biofilm formation and motility regulation*.

The majority of genes were downregulated in absence of ErsA (139 genes); the strongest negative effect was observed for *narK1* involved in nitrate transport. The other hits with a change of Log_2_(FC) ≤-1.5, comprise well described genes involved in biofilm formation and motility (*algD, esrC, ppyR, pelCDEFG, roeA*), energy and carbon metabolism (*prpD, prpC, coxA, coxB*), heat-shock proteins (*htpG, hslU, hslV, ibpA, dnaK, dnaJ*) and *phzS* involved in pyocyanin production.

### ErsA Binds *in Vitro* and Positively Regulates *in Vivo amrZ* mRNA at the Post-transcriptional Level

**FIGURE 4:**
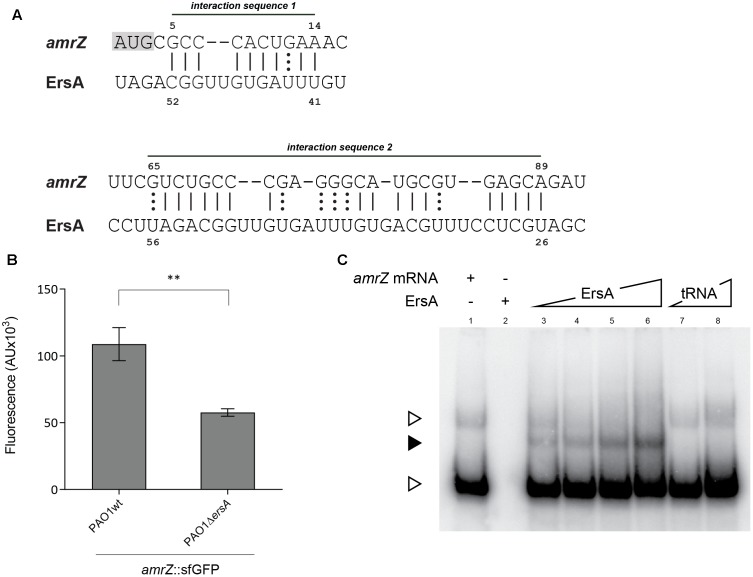
Interaction of ErsA with *amrZ* mRNA. **(A)** Prediction by IntaRNA software of the two base-pairing interactions between ErsA and *amrZ* mRNA. ErsA is predicted to bind to *amrZ* mRNA at two different sites in the ORF; the interaction sequence 1 is close to the ATG (highlighted in gray). **(B)** Comparison of the fluorescence polarization expressed in arbitrary units (AU) resulting from the translational fusion *amrZ*::*gfp* in PAO1 and PAO1 Δ*ersA*. The absence of ErsA results in a reduction of the reporter activity compared to the reference strain (statistical analysis performed on three individual clones per strain using *T*-test, ^∗∗^*p*-value < 0.01). **(C)** *In vitro* interaction between ErsA RNA and *amrZ* mRNA by electrophoretic mobility shift assay. Increasing amounts of ErsA RNA (0.15, 0.3, 0.6, and 1.2 pmol; lanes 3–6) or, as a negative control, yeast tRNA (0.89 and 8.9 pmol; lanes 7 and 8) were incubated with 0.3 pmol of *amrZ* mRNA at 37°C for 20 min and loaded onto a native 6% polyacrylamide gel. Nucleic acids were transferred onto Hybond N+ nylon membranes. After blots, the ErsA-mRNA interactions were tested using oligonucleotide probes for the mRNA target. Free target mRNA is indicated with open arrowheads, sRNA/mRNA complex with filled arrowheads.

In order to investigate the possibility that ErsA regulates biofilm modulating the expression of AmrZ at the post-transcriptional level through direct binding to the *amrZ* mRNA, we used a plasmid based GFP-reporter system and an electromobility-shift assay for the *in vivo* and *in vitro* validation, respectively. Before this, however, we used the full-length ErsA RNA sequence and the *amrZ* mRNA (including the 5′ untranslated region, 5′-UTR), as inputs in the web tool *IntaRNA* (Wright et al., 2014) to predict ErsA-*amrZ* mRNA interactions. The tool identified two putative interaction sites for ErsA on the *amrZ* mRNA. The interaction site 1 (IS1) involves part of the ErsA U-rich unstructured region, from nt 41 to 52 and is predicted to bind to *amrZ* mRNA in the region spanning +5 to +14 from the translational starting site AUG (**Figure 4A**). The ErsA interaction site 2 (IS2) on *amrZ* mRNA is predicted at positions +65 to +89 and covers a longer region on ErsA unstructured structure, from 26 to 56 nt (**Figure 4A**).

To test the ErsA post-transcriptional regulation on *amrZ* mRNA, we generated a translational fusion between the 5′-UTR along with the first 35 codons of *amrZ* mRNA and the superfolder variant gene of the green fluorescent protein (sfGFP) under the control of the heterologous constitutive promoter *P*_*LtetO*-1_. This GFP reporter fusion was transformed into *P. aeruginosa* PAO1 wild-type and Δ*ersA* strains, respectively. As shown in **Figure 4B**, the ErsA deletion caused a reduction in GFP activity of the *amrZ::sfGFP* translational fusion compared to the wild-type and it was possible to increase the *amrZ::sfGFP* translational levels in Δ*ersA* mutant strain by inducing with arabinose the expression of *ersA* from the pGM-*ersA* plasmid (Supplementary Figure S3), suggesting a direct effect of ErsA on *amrZ* translation efficiency. The lack of a full genetic complementation could be explained by the fact that we observed by Northern blot that the ErsA levels expressed by pGM-*ersA* in a Δ*ersA* strain are lower than those expressed by the chromosomal copy of *ersA* gene (data not shown). This scenario is different from the one observed for the expression of ErsA from pGM-*ersA* in a wild-type background where the ErsA levels resulted to be five–sixfold higher than those expressed by the chromosomal copy of *ersA* gene (Ferrara et al., 2015). This would suggest a higher ErsA degradation in a Δ*ersA* background.

Interactions of ErsA with the GFP ORF were previously controlled using a plasmid carrying exclusively the *gfp* gene (Ferrara et al., 2015). These results strongly suggested a positive regulation by ErsA on translation of the *amrZ* gene. This regulation does not depend on Hfq (data not shown). Furthermore, to document the predicted ErsA–*amrZ* mRNA interaction also *in vitro*, ErsA RNA and *amrZ* mRNA were synthesized, mixed and analyzed by electrophoresis on native polyacrylamide gels. As shown in **Figure 4C**, ErsA specifically formed a complex with the *amrZ* mRNA.

**FIGURE 5:**
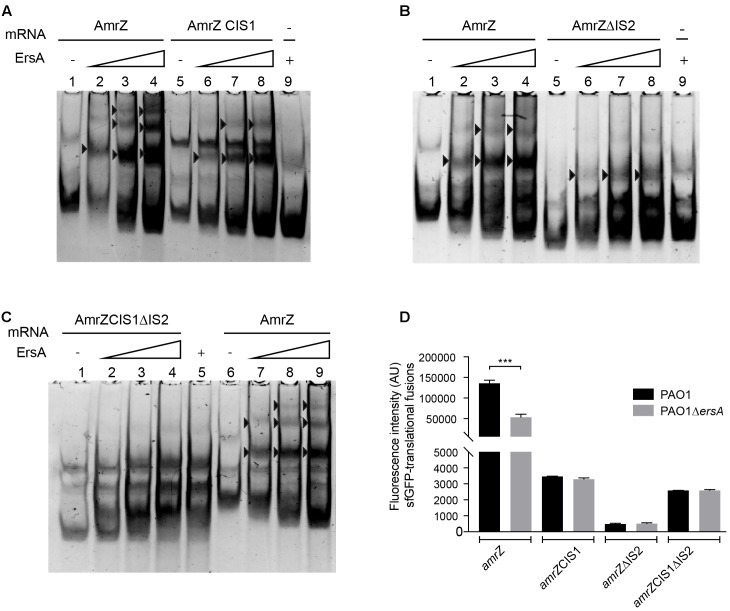
*In vitro* (non-radioactive EMSA) and *in vivo* analysis of ErsA interactions with *amrZ* and *amrZ* modified transcripts. **(A)** Interactions between ErsA-*amrZ* mRNA and ErsA- *amrZ*CIS1 mRNA generated by substitution of IS1 with its complement sequence. **(B)** Interactions between ErsA-*amrZ* mRNA and ErsA- *amrZ*ΔIS2 mRNA carrying the deletion of IS2. **(C)** Interactions between ErsA-*amrZ* mRNA and ErsA- *amrZ*CIS1ΔIS2 mRNA characterized by both the modifications present in *amrZ*CIS1 and *amrZ*ΔIS2. ErsA specifically binds *amrZ*, *amrZ*CIS1, and *amrZ*ΔIS2 mRNAs (black arrows) but no complex is formed when combined to *amrZ*CIS1ΔIS2 mRNA. Binding reactions were performed mixing the *amrZ* mRNAs (5 pmol) with increasing amount of ErsA RNA (ratio 1:0.5, 1:1, 1:2). ErsA RNA free form 10 pmol (**A** lane 9, **B** lane 9, **C** lane 5), *amrZ* mRNA free form 5 pmol (**A** lane 1, **B** lane 1, **C** lane 6). **(D)** Comparison of the fluorescence intensity expressed in arbitrary units (AU) deriving from *amrZ::sfGFP*, *amrZ*CIS1*::sfGFP*, *amrZ*ΔIS2*::sfGFP* and *amrZ*CIS1ΔIS2*::sfGFP* in PAO1 wild-type and Δ*ersA* strains. Modification in ErsA interaction site IS1 and/or IS2 causes a reduction in the translational levels of *amrZ* mRNA in PAO1 wild-type, comparable to those measured in *ersA* deleted strain. *T*-test ^∗∗∗^*p*-value < 0.001.

To further document the specific ErsA-*amrZ* mRNA interactions, we generated three *amrZ* mRNA fragments, (i) *amrZ*CIS1, in which the interaction site 1 has been substituted with its complementary sequence, (ii) *amrZ*ΔIS2 characterized by the deletion of the interaction site 2 and (iii) *amrZ* CIS1ΔIS2 containing both the modifications present in *amrZ*CIS1 and in *amrZ*ΔIS2. The *in vitro* analysis showed that ErsA forms a complex with both *amrZ*CIS1 and *amrZ*ΔIS2 (**Figures 5A,B**), and it does not bind to *amrZ* CIS1ΔIS2 mRNA (**Figure 5C**). This suggested that both interaction sequences are involved in ErsA-*amrZ* binding (Supplementary Figure S4). *In vitro* results were corroborated by *in vivo* experiments, measuring the translational levels of *amrZCIS1::sfGFP*, *amrZ*Δ*IS2::sfGFP* and *amrZCIS1*Δ*IS2::sfGFP* in PAO1 wild-type and Δ*ersA* strains. The absence of the interaction sites for ErsA causes a reduction of translational fusions activity in both genetic backgrounds (**Figure 5D**), associated also to a transcriptional instability (data not shown).

## Discussion

ErsA is a 132 nt long sRNA expressed in *P. aeruginosa* in concert with other stress-induced genes. We have previously reported that ErsA regulates exopolysaccharide production, negatively affecting at the post-transcriptional level *algC* mRNA translation in an incoherent feed-forward loop driven by the alternative sigma factor σ^22^ (Ferrara et al., 2015). Several sRNAs can regulate a broad spectrum of mRNA targets, usually governing similar or correlated cellular processes (Storz et al., 2011). In this work, we expanded the target spectrum of ErsA, validating its direct interactions with the transcriptional regulator AmrZ, which is involved in biofilm and motility, in particular by promoting multicellular colony formation and repressing swarming and twitching motility.

*Pseudomonas aeruginosa* strains exhibiting increased swarming phenotype generally develop flat and uniform biofilm in flow cell experiments (Shrout et al., 2006). Likewise, twitching motility is suggested to be required for monolayer creation during the initial stages of biofilm development (Shrout et al., 2006; Guttenplan and Kearns, 2013). In addition, in Gram-negative bacteria, biofilm formation and cellular motility are inversely regulated (O’Toole and Kolter, 1998; Wang et al., 2014). According to these observations, inactivation of *ersA* gene results in increased twitching and swarming motility leading to a less structured biofilm matrix resulting in development of homogeneous monolayers with high surface to volume ratios compared to the wild-type strain.

These phenotypes were supported by genome-wide expression analysis, showing that inactivation of ErsA affects expression of several genes involved in biofilm development and motility regulation, such as *pelCDEFG*, *algD*, *ppyR*, and *roeA*. All these genes are known to be directly or indirectly regulated by the transcriptional regulator AmrZ (Jones et al., 2014; Xu et al., 2016).

Small RNAs can positively or negatively affect translation of transcriptional regulators. For example, three sRNAs, DsrA, MicF, and GcvB, inhibit translation of the *lrp* gene, coding for a transcriptional regulator involved in amino acid transport and utilization (Ottesman et al., 1998; Majdalani et al., 2002; Massé et al., 2005; Prévost et al., 2007). The results of this work strongly suggest that ErsA positively affects *amrZ* translation through direct binding to *amrZ* mRNA at two different segments located on the mRNA, IS1 and IS2, with the former positioned close to the translational starting site. ErsA binds to these two regions with the same segment as involved in the *algC* interaction (Ferrara et al., 2015). Likewise ErsA, other sRNAs are known to regulate target expression via multiple interactions. SgrS, a regulator of the *manXYZ* operon binds two different sites, both involved in RNaseE-dependent degradation of the mRNA (Rice et al., 2012); the aforementioned GcvB sRNA, interacts with two independent regions on the *lrp* mRNA (Lee and Gottesman, 2016); and RyhB is suggested to repress expression of *msrB*, a methionine oxidase gene, interacting with two sites on the same mRNA (Bos et al., 2013).

It is possible that concomitant binding of two ErsA RNAs to the *amrZ* mRNA, is required to remodel *amrZ* mRNA secondary structure in order to release the AUG from the interaction with the anti-AUG sequence present in *amrZ* mRNA in its unbound form (Supplementary Figure S4). These interactions would expose the translational starting site and improve the efficiency of translation of *amrZ* transcript, thus explaining the positive contribution of ErsA at the post-transcriptional level.

Even though we identified biofilm genes being part of the AmrZ regulon and therefore differentially expressed in the absence of ErsA, the transcriptomics data does not reflect in all cases the known regulation exerted by AmrZ. For example, the *roeA* and *ppyR* genes, suggested to be positively regulated by ErsA, are known to be repressed by AmrZ (Sternberg et al., 2008; Merritt et al., 2010; Jones et al., 2014). We cannot exclude that ErsA may also stabilize directly these transcripts, for instance protecting them from degradation, or that these effects depend on the activity of other regulators affecting *roeA* and *ppyR* expression. Therefore, ErsA seems to overlap with the AmrZ regulon in guiding the switch from a motile life-style into the biofilm mode, extending our previous findings of its involvement in extracellular matrix production (Ferrara et al., 2015). ErsA, thus stimulates indirectly exopolysaccharide production through its control of AmrZ translation; acting on AlgC, it may redirect the sugar precursor fluxes providing more building blocks for extracellular polysaccharides biosynthesis (**Figure 1**). ErsA, in this sense, may be part of a mixed-regulatory circuit, like that involved in high osmolarity response in *E. coli* (Guillier et al., 2006).

This mixed-regulatory circuit could be used to take advantage of ErsA in order to have a more rapid and enhanced response compared to transcriptional regulators, in particular in stress conditions (Shimoni et al., 2007) or for niche-competition in case of mixed-species biofilms. Indeed, ErsA has recently been described to be overexpressed in *P. aeruginosa* biofilm grown with *Staphylococcus aureus*. However, the role of ErsA in neutralizing *S. aureus* agents has to be investigated (Miller et al., 2017).

Thus, ErsA may be employed as a “fast switcher” in the regulation of biofilm development at multiple stages and regulatory levels, fine-tuning the main routes controlled by the alternative sigma factor σ^22^ in the transition between acute and chronic infection of *P. aeruginosa.*

## Author Contributions

GB, SF, and SM conceived and designed the study. MF, SF, GB, and SM conceived the experiments. MF, SF, and ER designed and performed the experiments. MF, GB, SF, SM, ER, and HJ analyzed the data. GB, SM, and HJ contributed reagents, materials and analysis tools. MF, GB, and SM wrote the paper.

## Conflict of Interest Statement

The authors declare that the research was conducted in the absence of any commercial or financial relationships that could be construed as a potential conflict of interest. The reviewer CN and handling Editor declared their shared affiliation.

## Footnotes

1 www.comstat.dk

2 www.ebi.ac.uk/arrayexpress
