# Supplementary material for: The Small RNA ErsA of Pseudomonas aeruginosa Contributes to Biofilm Development and Motility through Post-transcriptional Modulation of AmrZ
Source: Front Microbiol. 2018 Feb 15;9:238. doi: 10.3389/fmicb.2018.00238 (PMC5819304; doi:10.3389/fmicb.2018.00238)
Supplement: Supplementary file 1 [file Table_1.PDF]

Table S1. Bacterial strains and plasmids used in this study

**Strains**	**Description**	**Construction**	**Reference**
Pseudomonas aeruginosa			
PAO1	Wild type		(Olson et al., 2000)
PAO1Δ*ersA*	Markerless Δ*ersA*		(Ferrara et al., 2015)
PAO1 miniTn7(Gm)P$_{rrnB1}$-*gfp*-a	Gm^r, Cm^r	PAO1wt containing the chromosomal insertion miniTn7(Gm)P$_{rrnB1}$-*gfp* located just downstream of the coding region of *glmS* gene.	This study
PAO1Δ*ersA* miniTn7(Gm)P$_{rrnB1}$-*gfp* a	Gm^r, Cm^r	PAO1 Δ*ersA* containing the chromosomal insertion miniTn7(Gm)P$_{rrnB1}$-*gfp* located just downstream of the coding region of *glmS* gene.	This study
Escherichia coli			
TOP10	*mcrA Δ(mrr-hsdRMS-mcrBC) φ80lacZDM15 ΔlacX74 deoR recA1 araD139 Δ(ara-leu)7697 galU galK rpsL endA1 nupG*		Invitrogen
DH5α	*fhuA2 lac(del)U169 phoA glnV44 Φ80' lacZ(del)M15 gyrA96 recA1 relA1 endA1 thi-1 hsdR17*		Invitrogen
**Plasmids**	**Description**	**Construction**	**Reference**
pXG10-SF	sfGFP reporter plasmid; *lacZ::gfp* under P$_{LtetO\text{-}1}$, Cm^r		(Corcoran et al., 2012)
pXG10-*amrZ::sfGFP*	pXG10-SF derivative; P$_{LtetO\text{-}1}$ -*amrZ::gfp*, Cm^r	contains PCR product using oligos #1 and #2	This study
pBBR1-MCS5	lacZ$_{\alpha}$, Gm^r		(Kovach et al., 1995)
pBBR1-*amrZ::sfGFP*	pBBR1-MCS5 derivative; P$_{LtetO\text{-}1}$-*amrZ::sfGFP*, Gm^r	contains PCR product using oligos #5 and #6	This study

pUCIDT-*amrZ* CIS1	Plasmid cloning vector containing the synthetic gene *amrZ* CIS1. Amp^r	Generated by IDT (integrated DNA technologies)	This study
pBK-miniTn7(Gm)P_{rrnB1}-*gfp*-a	P_{rrnB1}, Amp^r, Gm^r, Cm^r		(Lambertsen et al., 2004)
pRK600	Helper plasmid, RP4/RK2 conjugation system, ColE1, Cm^r		(Christensen et al., 1998)
pUX-BF13	R6K, RP4 *mob*, Amp^r		(Bao et al., 1991)
pGM931	pHERD20T derivative, araC/P_{BAD} - tΩ, Apr		(Ferrara et al., 2015)
pGM-*ersA*	pGM931 derivative, *ersA* under P_{BAD}, Apr		(Ferrara et al., 2015)

Bao, Y., Lies, D. P., Fu, H., and Roberts, G. P. (1991). An improved Tn7-based system for the single-copy insertion of cloned genes into chromosomes of gram-negative bacteria. *Gene* 109, 167–168. doi:10.1016/0378-1119(91)90604-A.

Christensen, B. B., Sternberg, C., Andersen, J. B., Eberl, L., Møller, S., Givskov, M., et al. (1998). Establishment of new genetic traits in a microbial biofilm community. *Appl. Environ. Microbiol.* 64, 2247–2255. doi:10.1128/IAI.68.6.3140-3146.2000.

Corcoran, C. P., Podkaminski, D., Papenfort, K., Urban, J. H., Hinton, J. C. D., and Vogel, J. (2012). Superfolder GFP reporters validate diverse new mRNA targets of the classic porin regulator, MicF RNA. *Mol. Microbiol.* 84, 428–445. doi:10.1111/j.1365-2958.2012.08031.x.

Ferrara, S., Carloni, S., Fulco, R., Falcone, M., Macchi, R., and Bertoni, G. (2015). Post-transcriptional regulation of the virulence-associated enzyme AlgC by the σ(22) -dependent small RNA ErsA of Pseudomonas aeruginosa. *Environ. Microbiol.* 17, 199–214. doi:10.1111/1462-2920.12590.

Kovach, M. E., Elzer, P. H., Hill, D. S., Robertson, G. T., Farris, M. A., Roop, R. M., et al. (1995). Four new derivatives of the broad-host-range cloning vector pBBR1MCS, carrying different antibiotic-resistance cassettes. *Gene* 166, 175–6.

Lambertsen, L., Sternberg, C., and Molin, S. (2004). Mini-Tn7 transposons for site-specific tagging of bacteria with fluorescent proteins. *Environ. Microbiol.* 6, 726–732. doi:10.1111/j.1462-2920.2004.00605.x.

Olson, M. V., Stover, C. K., Pham, X. Q., Erwin, A. L., Mizoguchi, S. D., Warrener, P., et al. (2000). Complete genome sequence of Pseudomonas aeruginosa PAO1, an opportunisticpathogen. *Nature* 406, 959–964. doi:10.1038/35023079.
