# Supplementary material for: The Small RNA ErsA of Pseudomonas aeruginosa Contributes to Biofilm Development and Motility through Post-transcriptional Modulation of AmrZ
Source: Front Microbiol. 2018 Feb 15;9:238. doi: 10.3389/fmicb.2018.00238 (PMC5819304; doi:10.3389/fmicb.2018.00238)
Supplement: Supplementary file 2 [file Table_2.PDF]

Table S2. Oligonucleotides used in this study. Lowercase: sites for restriction enzymes. Bold: T7 promoter sequence.

Oligo #	Oligo name	Sequence (5´→ 3´)	Application
1	AmrZ_NsiI_Fw	GTTTTatgcatGAGAACAATGAACGCTTC	Amplification of part of the 5' UTR and 35 codons of *amrZ* ORF with *NsiI/Nhe*I ends for cloning in frame with *sfGFP* in pXG10-SF vector
2	AmrZ_NheI_Rv	GTTTTgctagcAGCGACTTCTGCGATCTG	
3	AmrZ DIS2_NheI_Rv	GTTTTgctagcAACGACGAATTTGTCAGCGGT	Amplification of part of the 5' UTR and 21 codons of *amrZ* ORF to generate *amrZ* ΔIS2 with *NsiI/Nhe*I ends for cloning in frame with *sfGFP* in pXG10-SF vector
4	T7_AmrZ_Fw	**CTAATCGACTCACTATAGGG**GAGAACAATGAACG	Amplification of *amrZ* in fusion with RNA polymerase T7 promoter for *in vitro* transcription
5	AmrZ_Rv_EMSA	AGCGACTTCTGCGATCTG	
6	AmrZ ΔIS2_Rv_EMSA	AACGACGAATTTGTCAGCGGT	Combined to primer number 3 for amplification of *amrZ* ΔIS2 in fusion with RNA polymerase T7 promoter for *in vitro* transcription

7	T7_ErsA_Fw	**CTAATACGACTCACTATAGGG**CGAATGGCTTCTTGAGCC	Amplification of ErsA in fusion with RNA polymerase T7 promoter for *in vitro* transcription
8	ErsA_Rv_EMSA	AAAAAAAACCCCGAGCTTCGTATGGGGAG	
9	Ptet-O1_ClaI_Fw	GTTTTatgcatTCCCTATCAGTGATAGAG	Amplification of the fragment spanning from the $P_{LtetO\text{-}1}$ promoter to the stop codon of *gfp* of pXG10-*amrZ::*sf*gfp* with *Cla*I/*Xba*I ends for cloning in pBBR1-MCS5
10	sfGFP_TAA_XbaI_Rv	TGATGCCtctagaTTATTTGTAGAGCTC	
11	sfGFP_+96_r	TTGTGCCCATTAACATCACCATC	Reverse primer on *gfp* for verification of constructs
