# Supplementary material for: The Small RNA ErsA of Pseudomonas aeruginosa Contributes to Biofilm Development and Motility through Post-transcriptional Modulation of AmrZ
Source: Front Microbiol. 2018 Feb 15;9:238. doi: 10.3389/fmicb.2018.00238 (PMC5819304; doi:10.3389/fmicb.2018.00238)
Supplement: Supplementary file 4 [file Image_1.PDF]

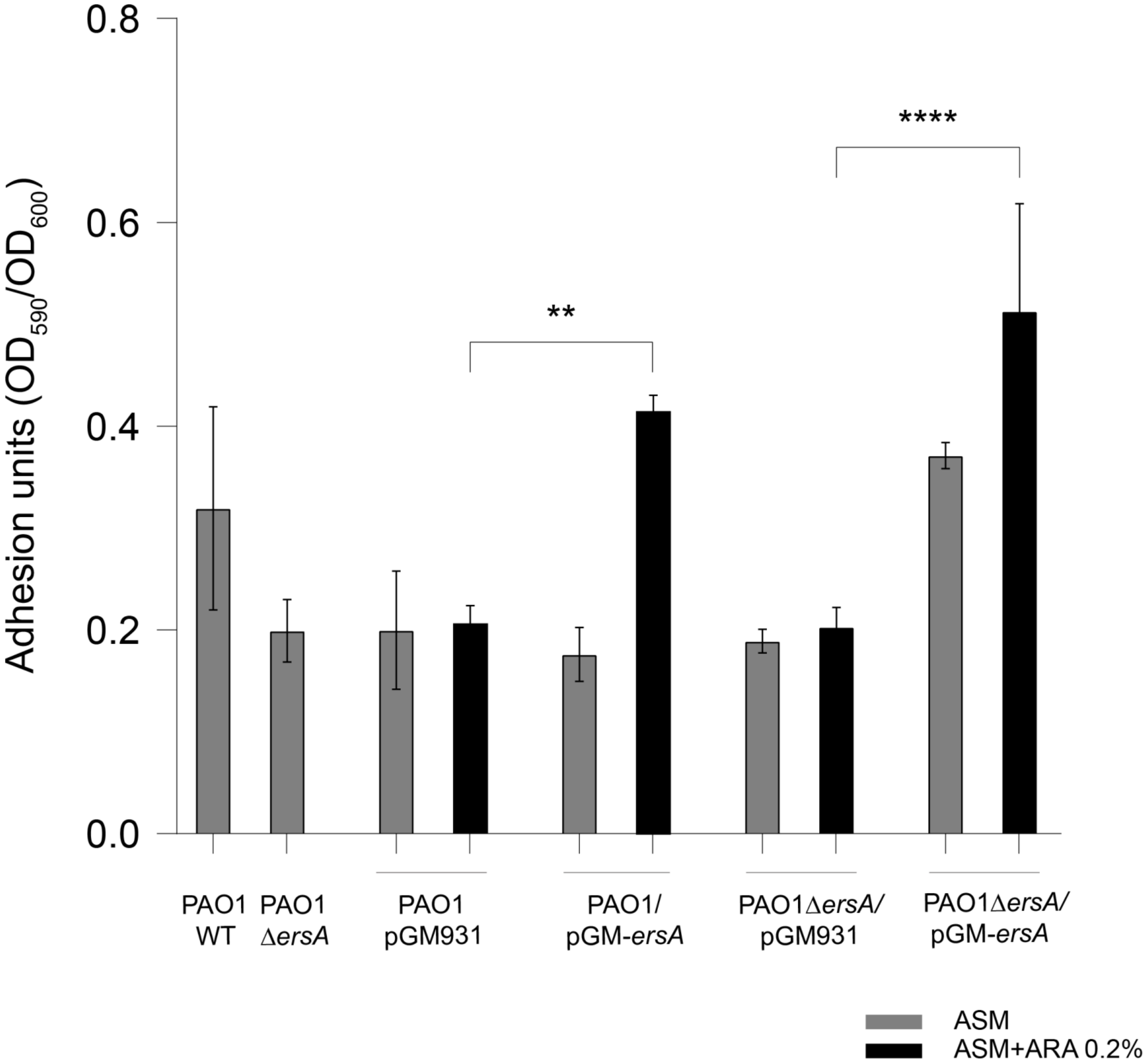

Figure S1. Biofilm assay in peg-lid microtiter. PAO1wild-type (WT) and Δ*ersA* background strains were grown in ASM at 37°C and 100 rpm shaking for 20h in presence of arabinose 0.2% to induce the *ersA* overexpression. The overexpression of ErsA induces an increment of biomass attached to the abiotic surface as quantified by crystal violet staining. The statistical analysis was performed using GraphPad Prism software (p.value < 0,01 **; < 0,001 ****).
