# Supplementary material for: The Small RNA ErsA of Pseudomonas aeruginosa Contributes to Biofilm Development and Motility through Post-transcriptional Modulation of AmrZ
Source: Front Microbiol. 2018 Feb 15;9:238. doi: 10.3389/fmicb.2018.00238 (PMC5819304; doi:10.3389/fmicb.2018.00238)
Supplement: Supplementary file 5 [file Image_2.PDF]

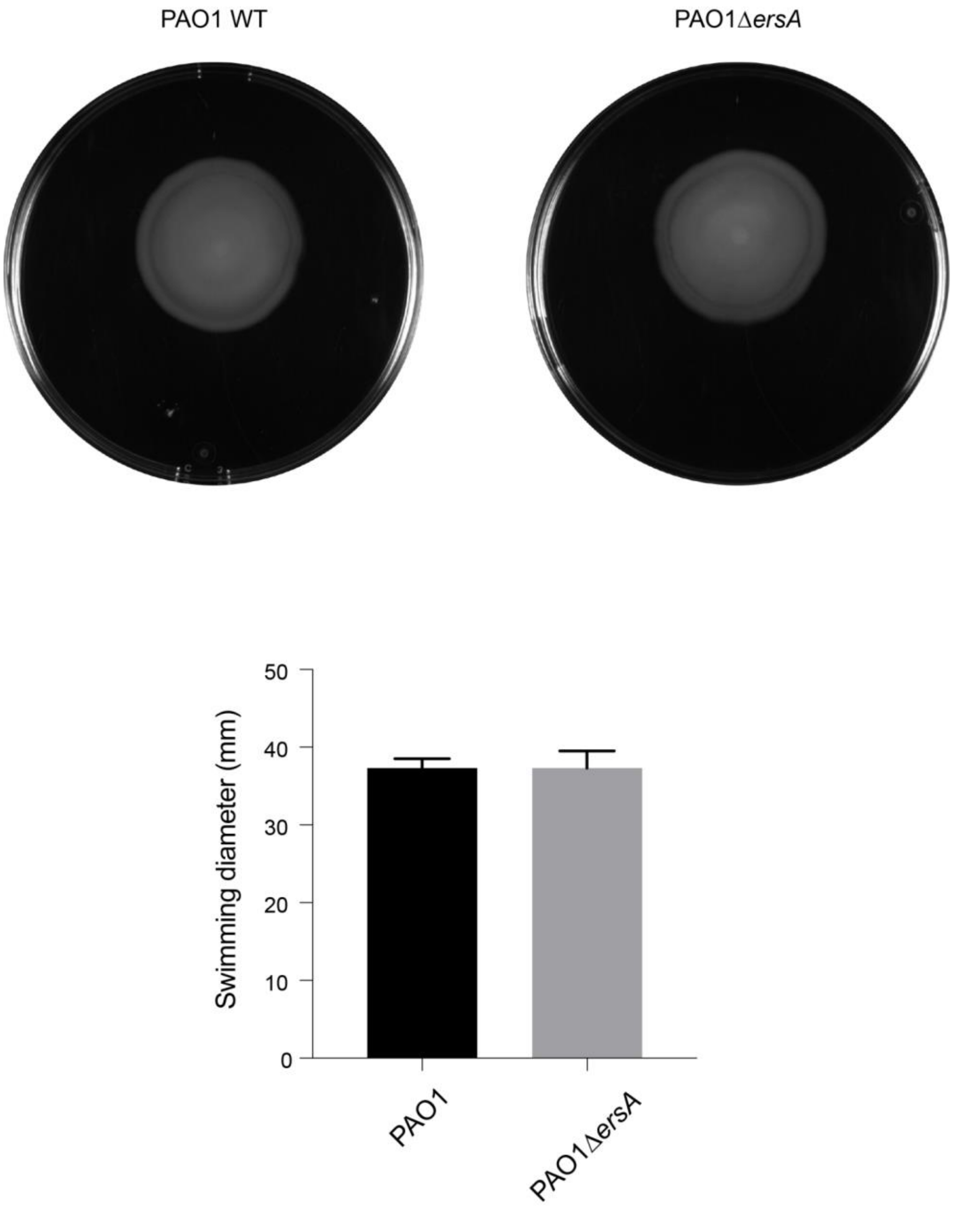

Figure S2. Swimming motility of PAO1 wild-type and Δ*ersA* strains performed in LB medium supplemented with agarose 0.3%, at 37°C for 18 hrs.
