# Supplementary material for: The Small RNA ErsA of Pseudomonas aeruginosa Contributes to Biofilm Development and Motility through Post-transcriptional Modulation of AmrZ
Source: Front Microbiol. 2018 Feb 15;9:238. doi: 10.3389/fmicb.2018.00238 (PMC5819304; doi:10.3389/fmicb.2018.00238)
Supplement: Supplementary file 6 [file Image_3.PDF]

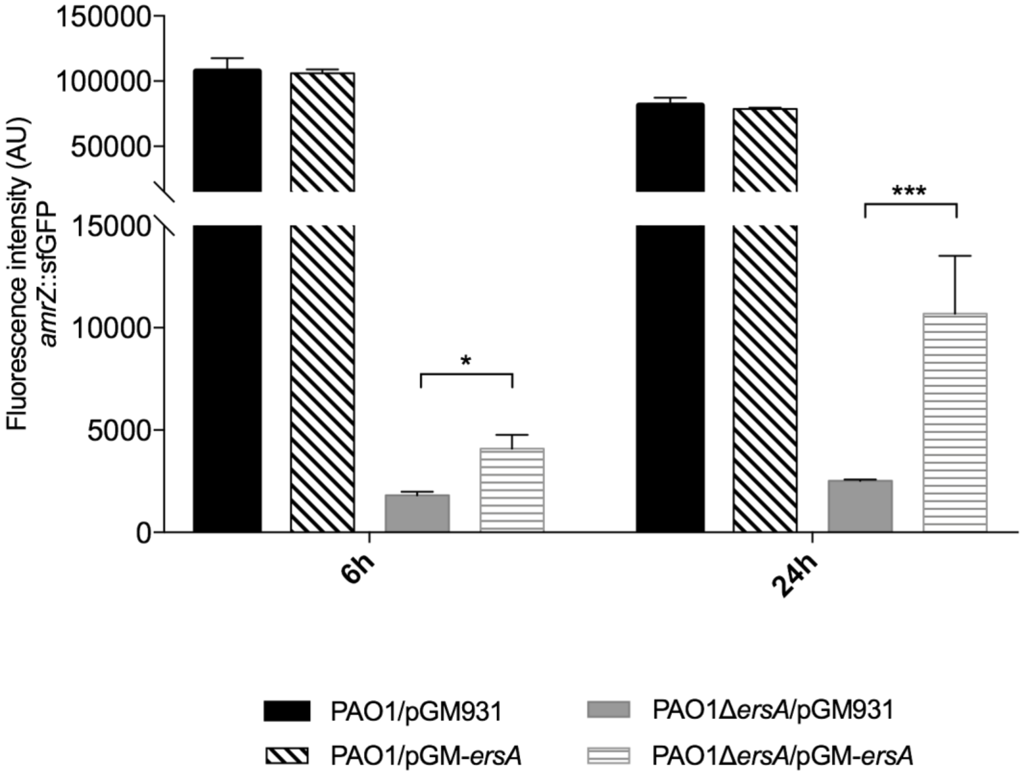

Figure S3. Comparison of fluorescence intensity of *amrZ::sfGFP* translational fusion combined with the control vector pGM931 and pGM-*ersA* in PAO1 wild-type and Δ*ersA* strains, in presence of arabinose 0.2% to induce *ersA* expression. Fluorescence intensity was measured after 6 and 24 hrs from induction with arabinose.
