# Supplementary material for: The Small RNA ErsA of Pseudomonas aeruginosa Contributes to Biofilm Development and Motility through Post-transcriptional Modulation of AmrZ
Source: Front Microbiol. 2018 Feb 15;9:238. doi: 10.3389/fmicb.2018.00238 (PMC5819304; doi:10.3389/fmicb.2018.00238)
Supplement: Supplementary file 7 [file Image_4.PDF]

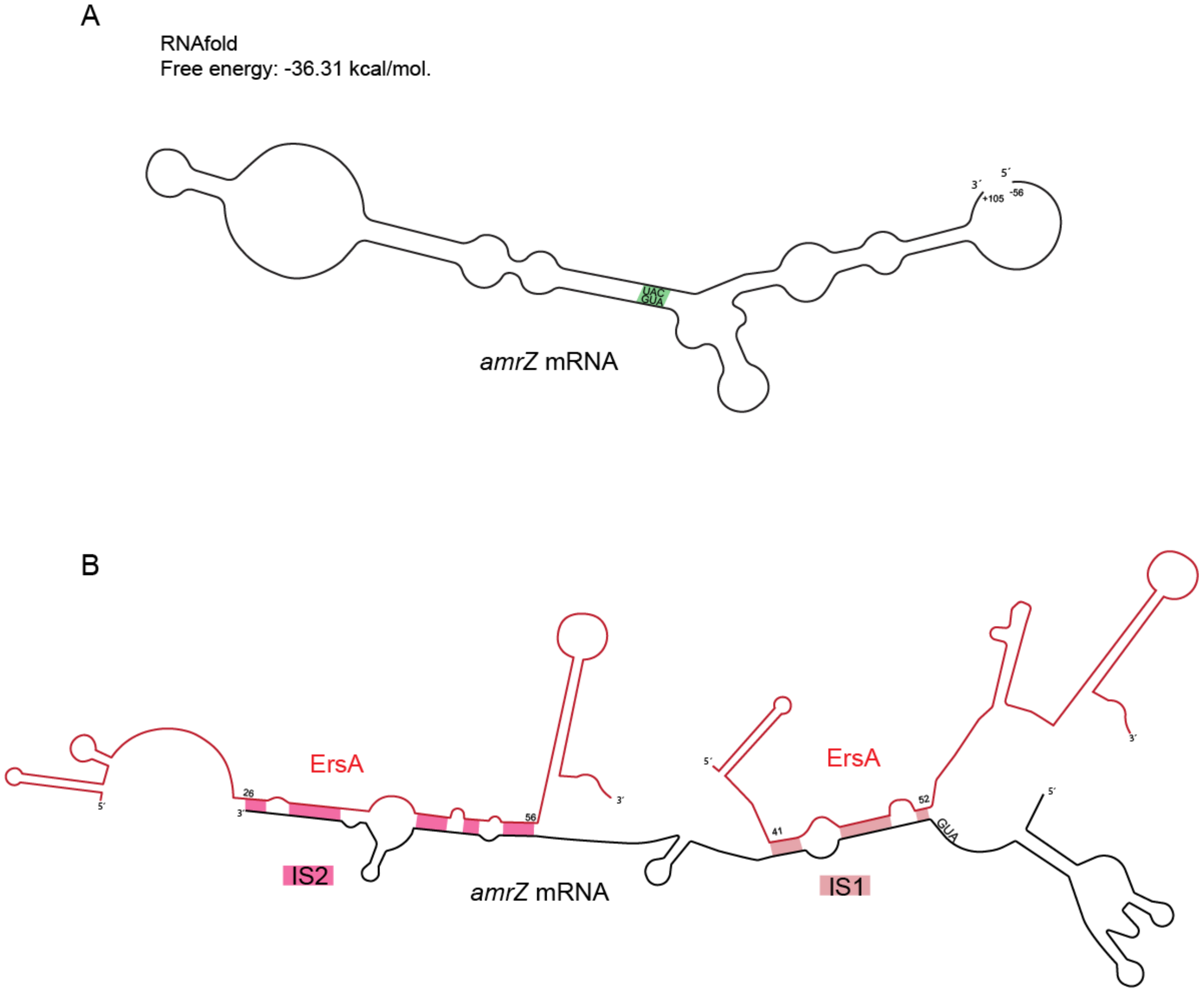

Figure S4. A. *amrZ* (from -56 to +105) secondary structure predicted by RNAfold software. **B.** Proposed model for ErsA-*amrZ* interaction. Two molecules of ErsA are suggested to bind *amrZ* mRNA in IS1 and IS2, remodeling the *amrZ* secondary structure and exposing the AUG.
